# Effect of hematoma volume on the 30-day mortality rate of patients with primary hypertensive brainstem hemorrhage: a retrospective cohort study

**DOI:** 10.3389/fsurg.2023.1136296

**Published:** 2023-05-05

**Authors:** Zhenxing Yu, Xianbin Zhang, Qiming Xu, Zhipeng Zhang, Yu Xia, Huaquan Li, Xiang Yu, Lei Deng, Long Zhang

**Affiliations:** Department of Neurosurgery, The 908th Hospital of the Joint Logistic Support of the People's Liberation Army, Nanchang, China

**Keywords:** hematoma volume, 30-day mortality, primary hypertensive brainstem hemorrhage, neurological damages, clinical signs and symptoms

## Abstract

**Objective:**

The purpose of this study is to investigate the effect of hematoma volume on the 30-Day Mortality Rate of patients with Primary Hypertensive Brainstem Hemorrhage (PHBH).

**Methods:**

Retrospective analysis was done on the clinical information of 74 patients who underwent treatment for primary hypertensive brainstem hemorrhage at the Department of Neurosurgery of the 908th Hospital of the Joint Logistic Support Force of the Chinese People's Liberation Army between January 2018 and December 2021. Both univariate and multivariate logistic regression were used to assess clinical signs and risk factors that affect 30-day mortality.

**Result(s):**

In the 74 patients with primary hypertensive brainstem hemorrhage included in this investigation, 46 patients died and 28 patients survived. The mortality rate at 30 days was 62.16%. A statistically significant difference was seen (*P* < 0.001) in the results of the univariate analysis, which suggested that hematoma volume may be a factor affecting the prognosis of patients with hypertensive brainstem hemorrhage. Hematoma volume was further demonstrated to be a risk factor and an independent factor impacting death in patients with brainstem hemorrhage (*P* < 0.001) by multivariate logistic regression analysis (OR: 2.6, 95% CI: 1.7–3.9, *P* < 0.001 Crude Model, OR: 3.6, 95% CI: 1.7–7.7, *P* < 0.001 Multivariate-Adjusted Model). After adjusting for confounding variables such as age, body mass index, sex, history of diabetes mellitus, history of hypertension, admission GCS score, stereotactic aspiration, combined hydrocephalus, admission systolic and diastolic blood pressure, the hematoma volume was revealed to be an independent predictor of 30-day death in patients with brainstem hemorrhage. We discovered by smooth curve fitting that hematoma volume increased in a non-linear manner with 30-day mortality. The 30-day mortality rate did not alter significantly when the hematoma volume was less than 4 ml. When the hematoma volume was greater than 4 ml, the 30-day mortality rate increased rapidly, and when the hematoma volume was 10 ml, the 30-day mortality rate reached the maximum.

**Conclusion(s):**

Hematoma volume is an independent factor affecting 30-day mortality in patients with primary hypertensive brainstem hemorrhage. The severe and extensive neurological damage caused by primary hypertensive brainstem hemorrhage is highly unlikely to be fundamentally altered by a single protocol, and new avenues need to be explored scientifically and continuously.

## Introduction

A spontaneous brainstem hemorrhage known as a primary hypertension brainstem hemorrhage (PHBH), which is unrelated to cavernous hemangioma, arteriovenous malformation, or any other disorders, is connected with hypertension. Primary hypertensive brainstem hemorrhage, which has an abrupt onset, quick progression, and a high fatality rate, is one of the most dangerous varieties of hemorrhagic stroke (1, 2). It primarily affects people between the ages of 40 and 60 and has a 2–4/100,000 yearly incidence rate, with men being more likely than women to be affected (3, 4). When a brainstem hemorrhage occurs, a hematoma typically develops quickly and frequently results in symptoms such as coma, tetraplegia, central fever, respiratory and circulatory failure, among others (5, 6). The hematoma caused by PHBH can quickly expand and result in a rapid deterioration of the patient's condition.

The management of PHBH remains controversial, and there is a need to better understand the factors that influence its prognosis (7). Previous research has shown that the volume of the hematoma is an independent predictor of the outcome in intracerebral hemorrhage (ICH), with a volume of 30 ml being a cutoff for death (8). However, the hematoma volume threshold for mortality outcomes in PHBH has not been adequately clarified. This study aims to investigate the clinical outcomes and risk variables that affect the prognosis of 74 patients with PHBH who received treatment in our department. The study uses a retrospective analysis of the patient's data to examine the relationship between hematoma volume and 30-day mortality in PHBH. Our hypothesis is that the threshold of hematoma volume is an independent predictor of 30-day death in patients with PHBH. By determining the threshold point of hematoma volume, this study aims to provide additional basis for optimizing treatment strategies for brainstem hemorrhage.

## Methods

### General information

**Figure 1:**
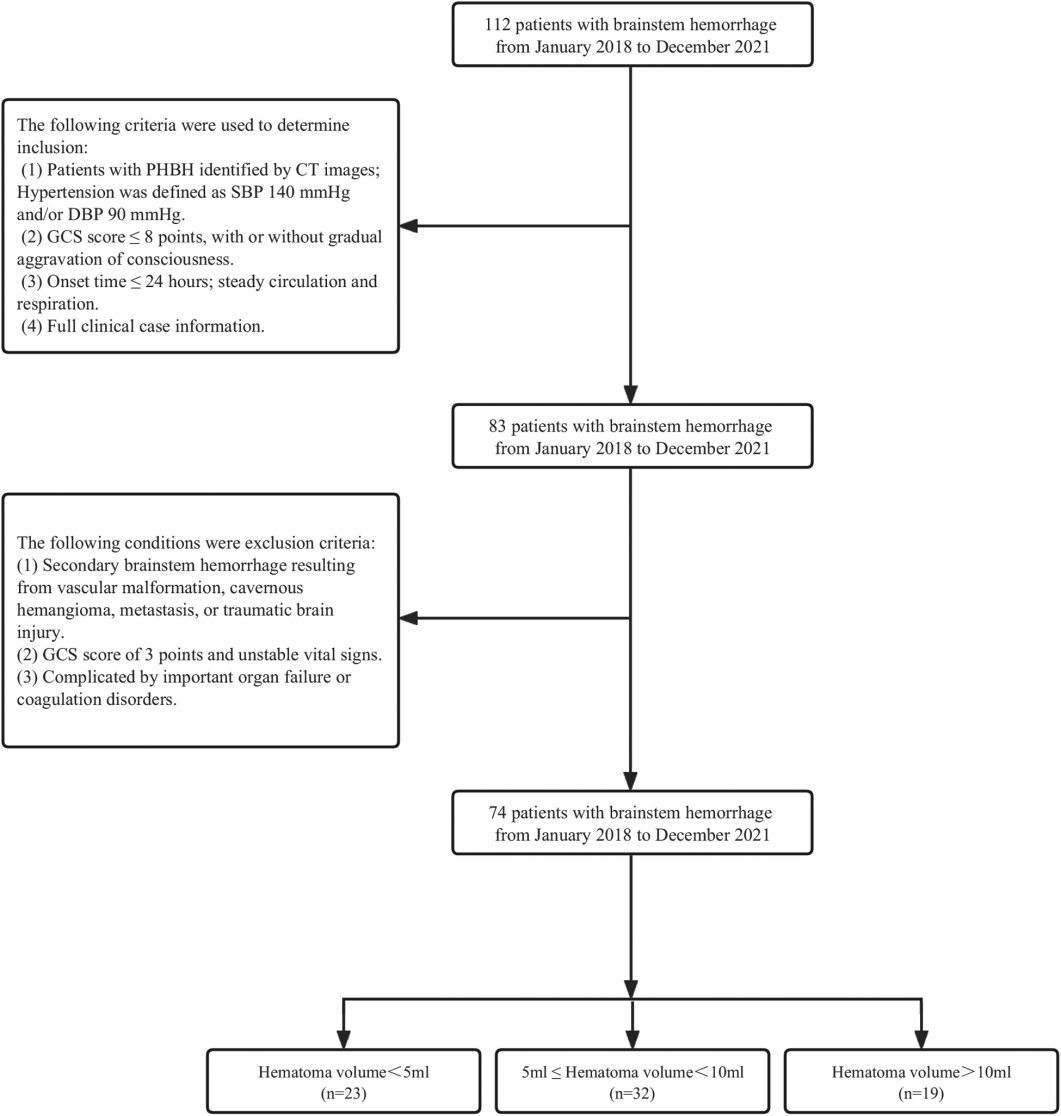
Flow chart of the study population selection and grouping.

This study comprised 74 PHBH patients who received care at the Department of Neurosurgery of The 908th Hospital of Joint Logistic Support Force of the Chinese People's Liberation Army between January 2018 and December 2021 (Figure 1). There were 28 females and 46 males, ranging in age from 30 to 80, with a mean age of 54.4. The Glasgow Coma Scale (GCS) indicated that 29 patients had 6–8 points and 45 cases had 3–5 points. This study was approved by the ethics committee of The 908th Hospital of Joint Logistic Support Force of Chinese People's Liberation Army, and the patients' families signed the informed consent. The data included in this report has never been published, in whole or in part, or offered for publication.

### Inclusion and exclusion criteria

The following criteria were used to determine inclusion: 1. Patients with PHBH identified by CT images; Hypertension was defined as SBP 140 mmHg and/or DBP 90 mmHg; 2. GCS score ≤8 points, with or without the gradual aggravation of consciousness; 3. Onset time ≤24 h; steady circulation and respiration; 4. Full clinical case information.

The following criteria were used to determine exclusion: 1. Secondary brainstem hemorrhage resulting from vascular malformation, metastasis, cavernous hemangioma, or traumatic brain injury; 2. Unstable vital signs and GCS score of 3 points; 3. Complicated by important coagulation disorders or organ failure.

### Imaging analysis

All 74 patients with brain computed tomography (CT) examinations had verified brainstem hemorrhage. The Tada formula (9) was used to compute the hematoma volume. All patients underwent a CT scan with a slice thickness of 5 mm, and the size and volume of the hematoma were calculated using the formula A × B × C/2, where A represents the diameter of the hematoma with the greatest diameter on the axial plane of the CT scan, B represents the diameter of the hematoma perpendicular to A on the same plane, and C represents the total length on the vertical plane (3, 10).

### Stereotactic aspiration surgery in PHBH

Equipment and instruments: We used a stereotactic frame to ensure accurate and consistent target localization. The surgical instruments included a high-speed drill with a side-cutting drill bit, a stereotactic biopsy cannula, and an aspiration catheter. A compatible stereotactic planning software was used to determine the coordinates for target localization based on preoperative CT scans.

Patient preparation: Prior to the procedure, the patient was placed under general anesthesia. The stereotactic frame was then fixed to the patient's head, ensuring both comfort and stability. Preopera-tive CT scans were acquired with the stereotactic frame in place, and these images were loaded into the stereotactic planning software to identify the coordinates of the target hematoma site.

Target localization: Using the stereotactic planning software, we calculated the target coordinates (x, y, z) and the entry angle for the surgical trajectory. The target site was chosen to minimize damage to critical structures, such as the brainstem and surrounding vasculature.

Surgical procedure: After sterilizing and draping the surgical site, a small skin incision was made at the entry point. The high-speed drill with a side-cutting drill bit was used to create a small burr hole in the skull. The stereotactic biopsy cannula was then carefully advanced through the burr hole, guided by the predetermined coordinates and trajectory angle. Once the tip of the cannula reached the hematoma, the aspiration catheter was inserted through the cannula, and the hematoma was gradually aspirated. Throughout the procedure, we carefully monitored the patient's neurological status and vital signs.

Postoperative management: Following the completion of the stereotactic drainage, postoperative CT scans were ob-tained to assess the reduction in hematoma volume and to monitor for any complications, such as rebleeding or infection. The patient was closely monitored in the intensive care unit (ICU) for any changes in neurological status or vital signs, and appropriate medical management was provided as needed.

### Efficacy evaluation

The prognosis of survivors was assessed using a modified Rankin scale (mRS) (11), where a score of mRS 3 points indicated a favorable prognosis and a score of mRS 4–6 points indicated bad prognosis (Table 1).

**Table 1:** The modified rankin scale (mRS).

Level	Details of the Modified Rankin Scale
0	No symptoms.
1	No significant disability. Able to carry out all usual activities, despite some symptoms.
2	Slight disability. Able to look after own affairs without assistance, but unable to carry out all previous activities.
3	Moderate disability. Requires some help, but able to walk unassisted.
4	Moderately severe disability. Unable to attend to own bodily needs without assistance, and unable to walk unassisted.
5	Severe disability. Requires constant nursing care and attention, bedridden, incontinent.
6	Dead.

### Outcome measures

Our research focused on each subject's vital status, which was categorized as either dead or living as a dichotomous variable. Readily available medical records were used to determine the date of death for the deceased participants. The following demographic, clinical data and 30-day mortality outcome data were analyzed: age, sex, past medical history, GCS score, stereotactic aspiration procedure, combined hydrocephalus, hematoma type, BMI, admission SBP, DBP, and hematoma volume. The hematoma volume was a continuous variable and was grouped into 3 groups according to less than 5 ml, 5–10 ml, and greater than 10 ml (12, 13). The primary outcome was defined as 30-day death. 30-day mortality was defined as a death that occurs within 30 days of the index event, usually admission, surgery, or medication in the hospital.

### Statistical analysis

The differences between the groups in terms of demographics, hematoma volume, and 30-day mortality were looked at. The mean and standard deviation were used to express continuous variables (SD). One-way ANOVA was used to compare continuous variables that had a normal distribution, while the Kruskal-Wallis test was used to analyze continuous variables with an atypical distribution. When comparing the distribution of categorical data, Pearson's chi-squared test or Fisher's exact test was used, depending on the situation. Categorical variables were presented as count and proportion. The probable causes of 30-day mortality were examined using univariate logistic regression analysis. To investigate the possible independent association between the hematoma volume and 30-day mortality, multivariate logistic regression analysis was used. As recommended by the STROBE guidelines, this study presents both unadjusted (Crude Model), slightly adjusted (Adjusted Model 1), and fully adjusted (Adjusted Model 2) equations. In the multivariable analysis, the confounding factor is an important issue, we performed some different statistical models to verify the results' stability. In the final model, we adjusted the factors basing the following three rules: 1. We adjusted for variables, if it was added to this model, the matched odds ratio would change by at least 10%. 2. For univariate analysis, we adjusted for variables, of which the *p* values <0.1. 3. For multivariable analysis, variables were chosen based on previous findings and clinical constraints. The results of these models are shown in (Table 5). The statistical software packages R (The R Foundation; version 4.2.0), and Empower (R) (X&Y solutions, Boston, MA, USA) were used for all statistical analyses. *P* < 0.05 was considered statistically significant.

## Results

### 30-Day mortality in patients with PHBH

Of the 74 patients with primary hypertensive brainstem hemorrhage treated in our department, 46 died and 28 survived. The 30-day mortality rate was 62.16% (Table 2).

**Table 2:** The 30-day mortality in patients with primary hypertensive brainstem hemorrhage.

	Number of cases (*n*)	Yes	No	Mortality rate (%)
30-Day mortality rate	*n* = 74	46	28	62.16

### Baseline characteristics

A total of 74 patients were divided into three groups based on hematoma volume (HV) as follows: HV < 5 ml (*n* = 23), 5 ≤ HV < 10 ml (*n* = 32), HV ≥ 10 ml (*n* = 19) (Table 3). Table 3 shows the comparison of baseline data, clinical indicators, and 30-day mortality for the 3 groups. No significant differences were observed in age, BMI, admission SBP, DBP, gender, and prior medical history among the different hematoma volume groups (all *p* values > 0.05). However, 30-day death increased significantly from the lowest HV < 5 ml group to the highest HV ≥ 10 ml group (P < 0.001).

**Table 3:** Baseline characteristics of participants (*N* = 74).

Hematoma Volume	T1	T2	T3	*P*-value
	HV < 5 ml	5 ≤ HV < 10 ml	HV ≥ 10 ml	
*N*	23	32	19	
Age, years	55.26 ± 12.12	56.59 ± 15.77	49.79 ± 14.41	0.256
BMI, kg/m^2^	22.39 ± 2.84	22.25 ± 2.90	23.16 ± 3.02	0.543
Admission SBP, mmHg	148.61 ± 12.39	157.91 ± 15.34	165.37 ± 21.06	0.005
Admission DBP, mmHg	87.04 ± 5.94	85.00 ± 7.82	85.21 ± 5.30	0.505
Gender				0.592
Female	10 (43.48%)	10 (31.25%)	8 (42.11%)	
Male	13 (56.52%)	22 (68.75%)	11 (57.89%)	
Prior medical history				0.814
History of hypertension	9 (39.13%)	18 (56.25%)	10 (52.63%)	
History of diabetes	1 (4.35%)	1 (3.12%)	0 (0.00%)	
Hypertension and diabetes	6 (26.09%)	8 (25.00%)	5 (26.32%)	
None	7 (30.43%)	5 (15.62%)	4 (21.05%)	
GCS score				0.011
6–8 points	14 (60.87%)	12 (37.50%)	3 (15.79%)	
3–5 points	9 (39.13%)	20 (62.50%)	16 (84.21%)	
Stereotactic aspiration surgery				0.017
No	7 (30.43%)	2 (6.25%)	1 (5.26%)	
Yes	16 (69.57%)	30 (93.75%)	18 (94.74%)	
Combined hydrocephalus				0.002
No	21 (95.45%)	24 (75.00%)	9 (47.37%)	
Yes	1 (4.55%)	8 (25.00%)	10 (52.63%)	
30-day mortality				<0.001
Survival	20 (86.96%)	8 (25.00%)	0 (0.00%)	
Dead	3 (13.04%)	24 (75.00%)	19 (100.00%)	
Hematoma type				<0.001
Massive type	1 (4.35%)	7 (21.88%)	5 (26.32%)	
Bilateral tegmental type	8 (34.78%)	19 (59.38%)	13 (68.42%)	
Basal tegmental type	9 (39.13%)	6 (18.75%)	1 (5.26%)	
Unilateral tegmental type	5 (21.74%)	0 (0.00%)	0 (0.00%)	

HV, hematoma volume; BMI, body mass index; SBP, systolic blood pressure; DBP, diastolic blood pressure; GCS, glasgow coma scale;.

### Univariate analysis of 30-Day mortality

Hematoma volume was found to have a significant impact on 30-day mortality by univariate logistic regression analysis (Table 4). Gender, age, hematoma type, prior medical history, BMI, and admission DBP all had a negative impact on 30-day mortality, while GCS score, stereotactic aspiration surgery, combined hydrocephalus, and admission SBP all had a positive impact.

**Table 4:** Univariate logistics regression analysis for 30-day mortality.

Covariate	OR (95%CI)	*P*-value
Age	0.98 (0.95, 1.01)	0.2332
Gender		
Female	Ref	
Male	1.41 (0.54, 3.69)	0.4880
Prior medical history		
History of hypertension	Ref	
History of diabetes	0.48 (0.03, 8.35)	0.6145
Hypertension and diabetes	0.82 (0.26, 2.62)	0.7416
None	0.48 (0.14, 1.59)	0.2297
GCS score		
6–8 points	Ref	
3–5 points	4.38 (1.60, 11.95)	0.0039
Stereotactic aspiration surgery		
No	Ref	
Yes	4.78 (1.12, 20.36)	0.0345
Combined hydrocephalus		
No	Ref	
Yes	7.89 (1.66, 37.54)	0.0094
Hematoma type		
Massive type	Ref	
Bilateral tegmental type	0.00 (0.00, _§)	0.9923
Basal tegmental type	0.00 (0.00, _§)	0.9912
Unilateral tegmental type	0.00 (0.00, _§)	0.9914
BMI	1.05 (0.89, 1.24)	0.5740
Admission SBP	1.05 (1.01, 1.08)	0.0060
Admission DBP	0.99 (0.92, 1.06)	0.6986
Hematoma volume	2.25 (1.60, 3.17)	<0.0001
Hematoma volume		
HV < 5 ml	Ref	
5 ≤ HV < 10 ml	20.00 (4.67, 85.57)	<0.0001
HV ≥ 10 ml	§. (0.00, _§)	0.9931

CI, confidence interval; OR, odds ratio; HV, hematoma volume; BMI, body mass index; SBP, systolic blood pressure; DBP, diastolic blood pressure; GCS: glasgow coma scale; § The model failed because of the small sample size.

### Multivariate logistic regression analysis for 30-day mortality

After adjusting for confounding variables such as age, sex, prior medical history, GCS score, stereotactic aspiration surgery, combined hydrocephalus, BMI, admission SBP, and DBP, hematoma volume was further examined using multivariate logistic regression. According to the findings, hematoma volume was a reliable predictor of 30-day death (Table 5). The odds ratio (OR) of 30-day mortality was 2.25 (95% CI: 1.60, 3.17), 2.26 (95% CI: 1.60, 3.20), and 2.90 (95% CI: 1.58, 5.32) in the crude model, adjusted model 1, and adjusted model 2, respectively, with statistically significant differences (*p* < 0.0001 and 0.0006, respectively). Furthermore, compared with the HV < 5 ml group, the 30-day mortality in the 5 ≤ HV < 10 ml group increased significantly (OR = 20.00 (95% CI: 4.67, 85.57), *P* < 0.0001, OR = 21.25 (95% CI: 4.74, 95.27), *P* < 0.0001 and OR = 32.00 (95% CI: 3.81, 268.58), *P* = 0.0014, respectively). Model failure for the HV ≥ 10 ml group was considered to be due to the small sample size in this group.

**Table 5:** Multivariate regression for effect of hematoma volume (ml) and 30-day mortality.

Outcome	Crude Model		Multivariate-Adjusted Model 1		Multivariate-Adjusted Model 2	
	*β*/OR (95%CI)	*P*-value	β/OR (95%CI)	*P*-value	β/OR (95%CI)	*P*-value
Hematoma volume	2.25 (1.60, 3.17)	<0.0001	2.26 (1.60, 3.20)	<0.0001	2.90 (1.58, 5.32)	0.0006
Hematoma volume						
HV < 5 ml	Ref		Ref		Ref	
5 ≤ HV < 10 ml	20.00 (4.67, 85.57)	<0.0001	21.25 (4.74, 95.27)	<0.0001	32.00 (3.81, 268.58)	0.0014
HV ≥ 10 ml	§. (0.00, _§)	0.9931	§. (0.00, _§)	0.9929	§. (0.00, _§)	0.9926

CI, confidence interval.

Crude Model: no adjustment.

Multivariate-Adjusted Model 1 adjusted for age and sex.

Multivariate-Adjusted Model 2 adjusted for age, sex, Prior medical history, GCS score, Stereotactic aspiration surgery, Combined hydrocephalus, BMI, Admission SBP, and DBP.

§ The model failed because of the small sample size.

### Smooth curve fitting analysis for 30-day mortality

**Figure 2:**
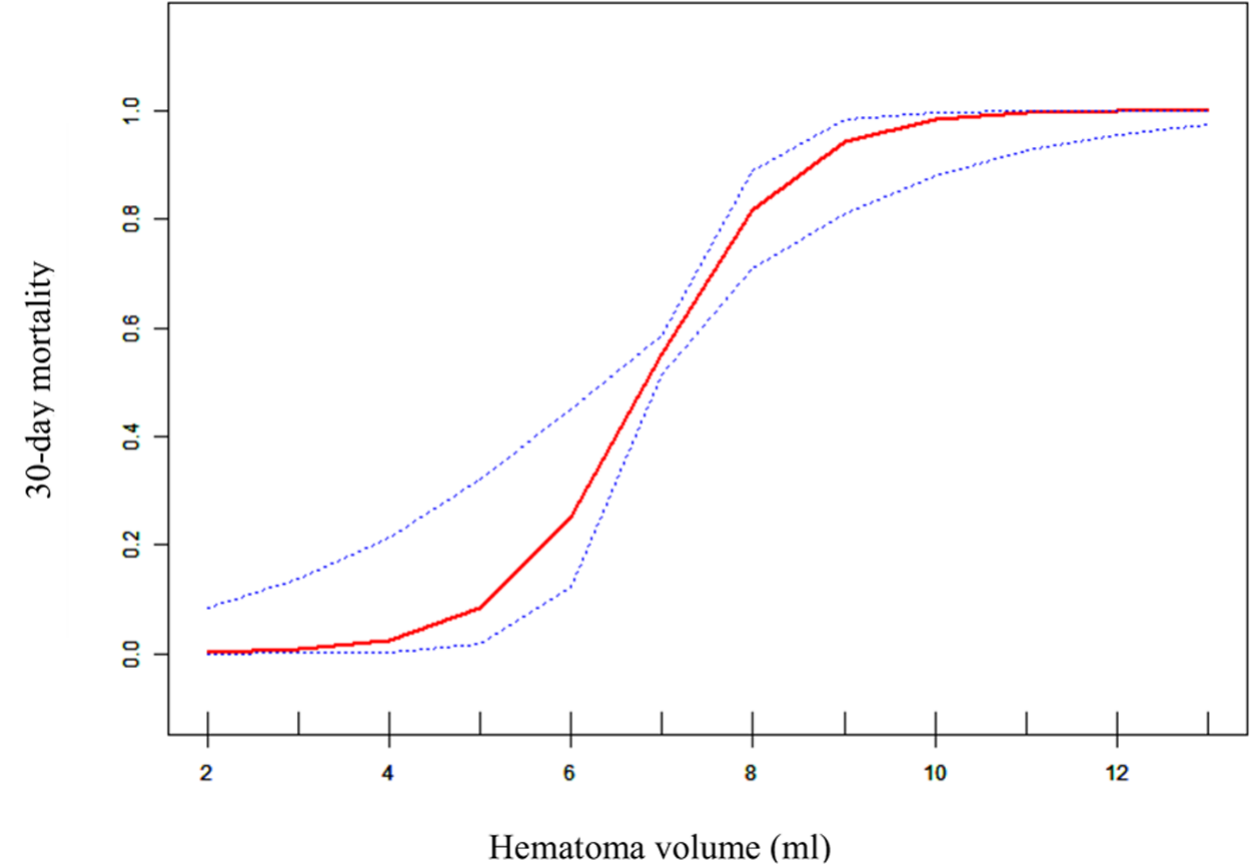
Curve fitting diagram of hematoma volume and 30-day mortality. A threshold, nonlinear association between hematoma volume and 30-day mortality was found in a generalized additive model (GAM). Solid rad line represents the smooth curve fit between variables. Blue bands represent the 95% of confidence interval from the fit. All adjusted for age, sex, prior medical history, GCS score, Stereotactic aspiration surgery, combined hydrocephalus, BMI, Admission SBP and DBP.

To evaluate the non-linear relationship between hematoma volume and the 30-day mortality, a fitting curve was applied (Figure 2). The result of smooth curve fitting revealed a non-linear relationship between hematoma volume and 30-day mortality after adjusting for age, sex, prior medical history, GCS score, stereotactic aspiration surgery, combined hydrocephalus, BMI, admission SBP, and DBP. As the hematoma volume increased, the 30-day mortality showed a trend of slight increase followed by a sharp increase and then saturation.

### Preoperative and postoperative imaging

**Figure 3:**
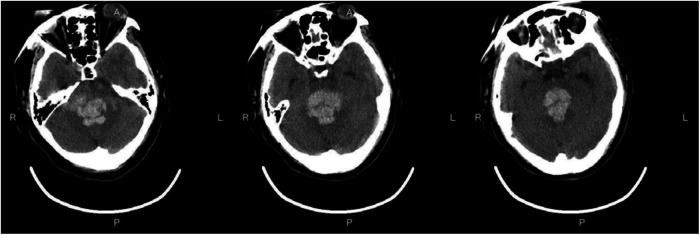
Preoperative imaging: Figure 3 shows a representative preoperative CT scan of a patient with PHBH.

**Figure 4:**
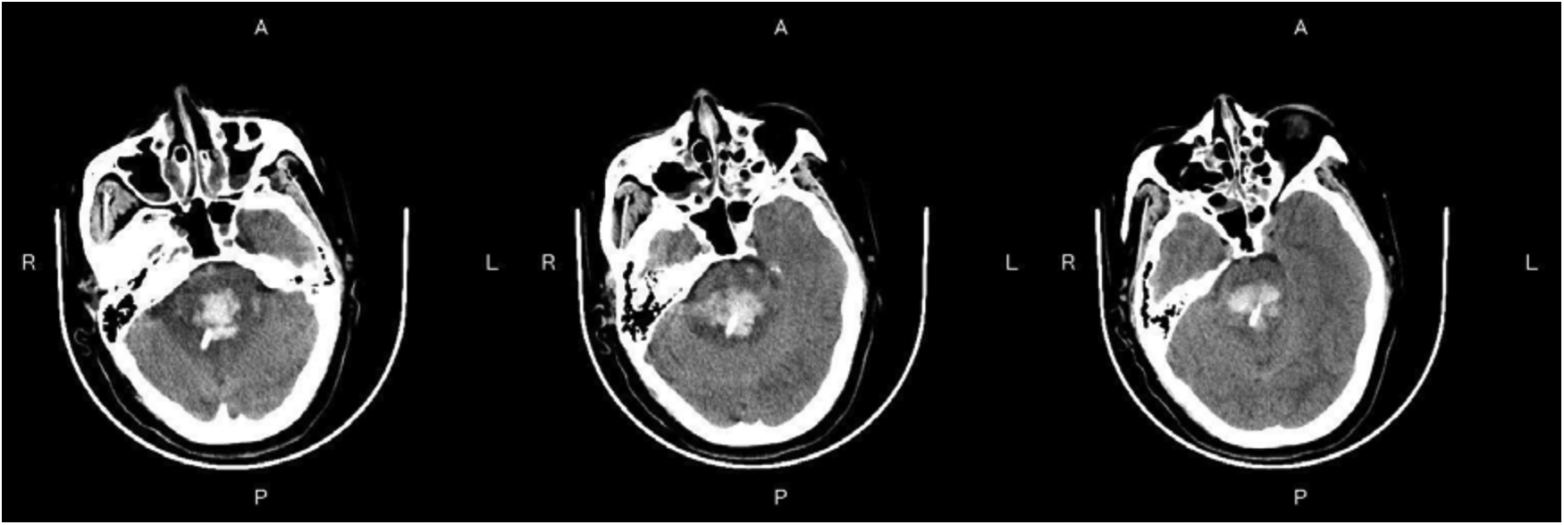
Postoperative imaging: Figure 4 presents a postoperative CT scan of the same patient, obtained 24 h after the stereotactic drainage procedure.

**Figure 5:**
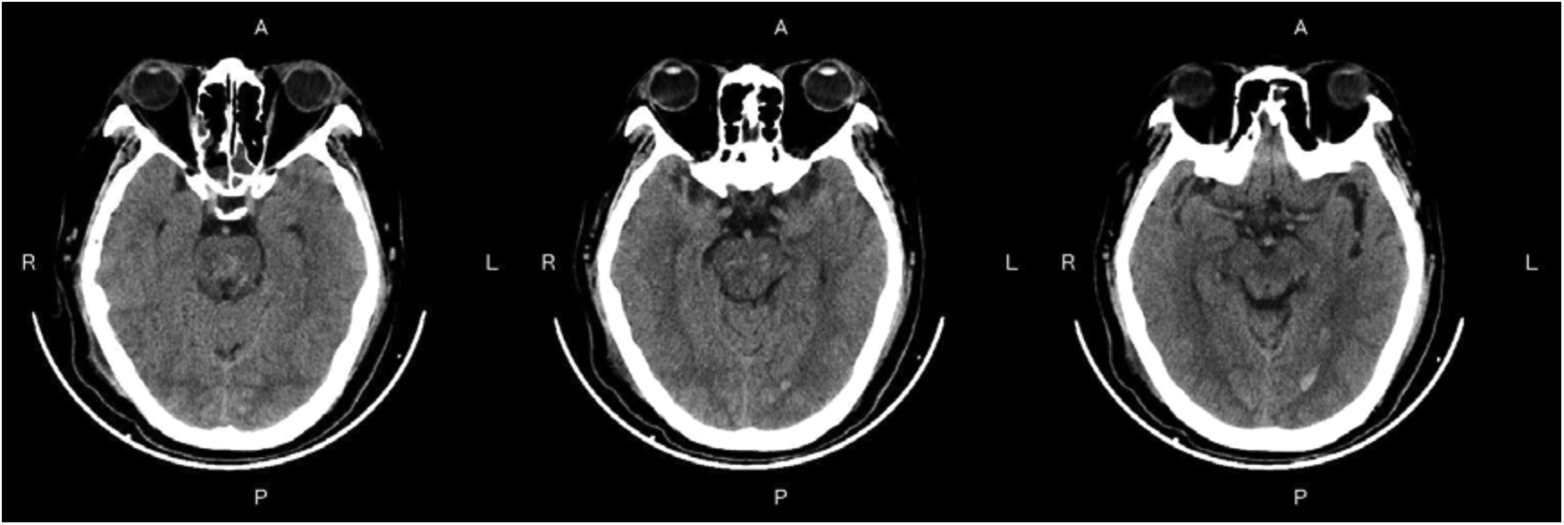
Postoperative imaging: Figure 5 presents a postoperative CT scan of the same patient, obtained 15 days after the stereotactic aspiration procedure.

Figure 3—Preoperative Imaging: Figure 3 shows a representative preoperative CT scan of a patient with PHBH. The image demonstrates a hyperdense hematoma in the brainstem region, causing compression of adjacent structures. Figure 4—Postoperative Imaging: Figure 4 presents a postoperative CT scan of the same patient, obtained 24 h after the stereotactic aspiration procedure. The image shows a significant reduction in the hema-toma volume, resulting in decreased mass effect and improved anatomical alignment of the brainstem and surrounding structures. Figure 5—Postoperative Imaging: Figure 5 presents a postoperative CT scan of the same patient, obtained 15 days after the stereo-tactic drainage procedure. The image shows the hematoma in brainstem is largely absorbed. These images, along with their accompanying descriptions and analyses, provide visual evidence of the effectiveness of the stereotactic drainage technique in reducing hematoma volume and alleviating mass effect in patients with PHBH.

## Discussion

In this retrospective cohort study, hematoma volume was an independent predictor of 30-day mortality in patients with primary hypertensive brainstem hemorrhage. This suggests that hematoma volume may be a very valuable parameter that could provide additional evidentiary support for optimizing treatment strategies. In this study, we also had a surprise finding. There was a nonlinear relationship between hematoma volume and 30-day mortality. When the hematoma volume was less than about 4 ml, the patient's 30-day mortality did not appear to change significantly. And when the hematoma volume was greater than about 4 ml, the patient's 30-day mortality appeared elevated and then rapidly and sharply increased, and when the hematoma volume reached about 10 ml, the 30-day mortality approached saturation. This indicated that the threshold point for hematoma volume and 30-day mortality may be around 4 ml, while the saturation point may be around 10 ml. To our knowledge, this is the first retrospective cohort study to reveal a nonlinear relationship between hematoma volume and 30-day mortality in patients with brainstem hemorrhage.

The two most frequent factors that induce brainstem bleeding are hypertension and atherosclerosis. According to earlier research, brainstem hemorrhage may produce the following pathological injuries. Firstly, the primary harm to the brainstem is generated by the mechanical destruction of hematoma; Secondly, the secondary injury is caused by the compression of hematoma, which results in local tissue ischemia, edoema, or an inflammatory response brought on by hematoma catabolic products (14). It has previously been established that the necrosis of brain tissue surrounding the hematoma starts within six hours of the initiation of intracerebral bleeding, intensifies after twelve hours, and reaches its peak within twenty-four hours (15). As a result, it is crucial to remove the brainstem hematoma as soon as possible following the onset of PHBH in order to minimize primary damage and avoid secondary damage, which is beneficial for maintaining brainstem nerve function (14).

In previous clinical studies of cerebral hemorrhage, brainstem hemorrhage was often excluded, and the guidelines of the American Heart Association (AHA)/American Stroke Association (ASA) did not recommend or even oppose brainstem hematoma evacuation (16). The results of several studies on PHBH in recent years have shown that minimally invasive surgery has a better prognosis than conservative treatment, and active clinical studies have been conducted. The primary surgical approaches for treating PHBH involve hematoma clearing via craniotomy and stereotaxis and/or navigation-guided hematoma puncture and drainage (2, 14). Some patients may benefit from stereotaxis or navigation-guided puncture of brainstem hematoma, according to early research. However, the hematoma cannot be totally cleared with such an approach, and once the bleeding begins, it cannot be halted under direct eyesight, restricting its application (17, 18). A common surgical procedure for the treatment of PHBH is the removal of hematomas by craniotomies, which can be carried out under direct vision while providing simultaneous accurate hemostatic and decompressive effects (2). However, the control of iatrogenic injury is still extremely challenging, and it needs to be carried out by experienced neurosurgeons, and attention should be paid to the operating principles of “no traction of the brainstem, light suction of hematoma, and weak electrocoagulation of the responsible vessel”. In this study, we followed Brown's Rule, multiplanar reformatting of brain CT scanning was combined, and individualized surgical approaches were adopted (19).

In this retrospective cohort study, 74 patients with PHBH were included for analysis. 64 patients underwent stereotactic aspiration and 10 patients were treated conservatively with medication. 30-day mortality was 62.16%. Patients who have experienced a brainstem hemorrhage may not benefit from surgical therapy, according to Fewel and Manno's research (20, 21). Surgical intervention was linked to 30-day mortality in Jang's research (6), however, Kearns et al. and Hara et al. argued that surgical intervention was effective (22, 23). This study's 30-day death rate of 62.16% was higher than earlier reports in the literature. We speculate that this may be due to differences in the study population, study design, etc. Surgical treatment for PHBH is a controversial issue, with some studies showing better outcomes with surgery and others showing no difference compared to conservative treatment. In light of this, the 30-day mortality rate for patients treated with surgery was compared to those not treated with surgery. The results showed a 30-day mortality rate of 59.37% for 64 patients treated with surgery and 80% for 10 patients not treated with surgery (Table 6). This suggests that surgical treatment may have a positive impact on 30-day mortality in PHBH patients, although further research is needed to confirm this. Nevertheless, we are optimistic about the surgical treatment of PHBH. With the popularization of observation equipment and the advancement of surgical techniques, the anatomical understanding of the safety zone of the brainstem has been improved, and some success has been achieved in removing the hematoma by selecting a suitable access route via the safety zone into the brainstem according to the location of the hematoma. We believe that the key factors influencing surgical choice are hematoma volume and hematoma type. In order to lower local and overall intracranial pressure and preserve CSF circulation, the surgical procedure should attempt to remove as much of the clot as feasible while causing the least amount of disturbance to the surrounding brain (24). Direct hematoma evacuation and successful decompression are two benefits of surgery. Nonetheless, there is a potential for increased risk from operation-induced tissue injury. According to our perspective, this operation is suitable for patients who have brain stem compression and who are unable to endure the possibility of a fourth ventricle obstruction brought on by a hematoma that has extended into the fourth ventricle. The right patient selection is one of the most crucial aspects impacting the outcome of surgery, and in our experience, the criteria are as follows. GCS ≤ 7 points for hematoma volume ≥5 ml; GCS ≤ 5 points for hematoma volume 3–5 ml, conservative until the onset of disease more than 72 h, or complicated by acute hydrocephalus.

**Table 6:** The 30-day mortality in patients with and without surgical treatment for PHBH.

Treatment	Number of cases (*n*)	Died within 30 days	Survived within 30 days	30-day Mortality Rate(%)
Surgery	*n* = 64	38	26	59.37
No Surgery	*n* = 10	8	2	80
Total	74	46	28	

Currently, there is plenty of research on the prognosis and affecting variables of hypertensive supratentorial hemorrhage (25, 26). However, there is a lack of research on brainstem hemorrhages. According to Raison et al., the location of the hematoma, the severity of the hemorrhage, and the patient's state at the time of onset al.l affect the prognosis of PHBH patients. The mortality rate approaches 100% when the hematoma volume is more than 10 ml (27, 28). The results of our study showed a mortality rate of 100.% (19/0) in patients with a hematoma volume greater than 10 ml and a mortality rate of 75% (24/32) in patients with a hematoma volume of 5–10 ml. The results of the univariate analysis revealed a significant difference in the impact of hematoma volume on prognosis [OR = 2.25 (95% CI: 1.60, 3.17), *P* < 0.0001], indicating that hematoma volume may play a role in determining the prognosis of brainstem hemorrhage. According to multivariate logistic regression analysis, the hematoma volume was not only a risk factor for death in patients with brainstem hemorrhage [OR = 2.90 (95% CI: 1.58, 5.32), P < 0.05], but it was also an independent factor (*P* = 0.0006). The findings were in line with those that had previously been published in the literature (7). Other studies have suggested that GCS score can be an independent risk factor for death in PHBH. In the present study, univariate analysis showed that the GCS score was statistically significant for 30-day death in patients with brainstem hemorrhage (*P* = 0.0039), which is consistent with previous reports in the literature (29). Meng's study showed no statistically significant effect of GCS score on the prognosis of patients with brainstem hemorrhage (*P* = 1.000). However, our study doesn't support these findings. In their study, many confounding factors, including age, sex, prior medical history, GCS score, stereotactic aspiration surgery, and combined hydrocephalus were not adjusted for. This may have biased their findings. Previous studies have shown that approximately 39.5% of PHBH entered the ventricular system, and the incidence of hydrocephalus was as high as 30.3% when the hemorrhage was located close to the fourth ventricle and midbrain aqueduct, with a markedly elevated risk of death and a poor prognosis (2, 30). In our study, we found that the presence of hydrocephalus was associated with 30-day mortality (*P* = 0.0094), which is consistent with previous results described in the literature. The fact that men were much more impacted by brainstem hemorrhage than women was also confirmed; However, we could not find any evidence of a gender or age difference (*P* = 0.592 and *P* = 0.256, respectively), which was consistent with the literature (30).

There are several benefits to our study. Firstly, in addition to using the generalized linear model to assess the linear association between hematoma volume and 30-Day mortality, we also utilize the generalized additive model to elucidate the nonlinear relationship. GAM has clear advantages when dealing with non-linear relations; it can handle non-parametric smoothing; and it will fit a regression spline to the data. We shall learn more about the true connections between exposure and outcome with the aid of GAM. Secondly, because this was an observational study with potential confounding that could not be avoided, we strictly applied statistical correction to reduce residual confounding. Even though the prior study found a linear relationship between hematoma volume and 30-Day mortality, we were unable to find this connection in our study even after adjusting for age, sex, prior medical history, GCS score, stereotactic aspiration surgery, combined hydrocephalus, BMI, admission SBP, admission DBP, and other confounding factors that the prior study had not taken into account.

## Limitation

This study has several restrictions. Firstly, the sample size of 74 patients in our study is relatively small, which may limit the statistical power of our analyses and the generalizability of our findings. The rarity of primary hypertensive brainstem hemorrhage may have contributed to the small sample size, as we had to rely on data from patients who were treated at our institution during a specific period. In addition, we did not perform a power analysis or an *a priori* sample size analysis, which may have further contributed to the small sample size. Secondly, the limitations of the retrospective cohort study design and the potential sources of bias and variance may have affected our analyses. We recognize that the retrospective design may have limited our ability to control for confounding variables and may have introduced selection bias or measurement bias. This study was limited to Chinese people, with certain regional and ethnic restrictions. In future studies, we will consider multi-center collaborations or expanding the scope of the study to include additional outcome measures or patient populations.

## Conclusion

Hematoma volume is an independent factor affecting 30-day mortality in patients with primary hypertensive brainstem hemorrhage. The severe and extensive neurological damage caused by primary hypertensive brainstem hemorrhage is highly unlikely to be fundamentally altered by a single protocol, and new avenues need to be explored scientifically and continuously. Future studies could build on these findings by exploring the relationship between hematoma volume and mortality further, investigating other factors that may contribute to mortality in these patients, and exploring new treatment strategies. By doing so, we may be able to develop more effective approaches to the management of primary hypertensive brainstem hemorrhage and ultimately improve outcomes for these patients.

## Data Availability

The raw data supporting the conclusions of this article will be made available by the authors, without undue reservation.
